# Scale-dependent diffusion anisotropy in nanoporous silicon

**DOI:** 10.1038/srep40207

**Published:** 2017-01-20

**Authors:** Daria Kondrashova, Alexander Lauerer, Dirk Mehlhorn, Hervé Jobic, Armin Feldhoff, Matthias Thommes, Dipanjan Chakraborty, Cedric Gommes, Jovana Zecevic, Petra de Jongh, Armin Bunde, Jörg Kärger, Rustem Valiullin

**Affiliations:** 1 University of Leipzig, Faculty of Physics and Earth Sciences, Linnéstraße 5, D-04103 Leipzig, Germany; 2 University of Gießen, Institute of Theoretical Physics, Heinrich-Buff-Ring 16, D-35392 Gießen, Germany; 3 Institut de Recherches sur la Catalyse et l’Environnement - CNRS 2, Avenue Albert-Einstein, F-69626 Villeurbanne Cedex, France; 4 Leibniz University Hannover, Institute of Physical Chemistry and Electrochemistry, Callinstr. 3-3A, D-30167 Hannover, Germany; 5 Quantachrome Ins., 1900 Corporate Drive, Boynton Beach, Florida 33426, USA; 6 Indian Institute of Science Education & Research Mohali, Sec 81, SAS Nagar, Manauli - 140306, Punjab, India; 7 Utrecht University, Department of Inorganic Chemistry and Catalysis, Sorbonnelaan 16, NL-3584 CA Utrecht, The Netherlands

## Abstract

Nanoporous silicon produced by electrochemical etching of highly B-doped p-type silicon wafers can be prepared with tubular pores imbedded in a silicon matrix. Such materials have found many technological applications and provide a useful model system for studying phase transitions under confinement. This paper reports a joint experimental and simulation study of diffusion in such materials, covering displacements from molecular dimensions up to tens of micrometers with carefully selected probe molecules. In addition to mass transfer through the channels, diffusion (at much smaller rates) is also found to occur in directions perpendicular to the channels, thus providing clear evidence of connectivity. With increasing displacements, propagation in both axial and transversal directions is progressively retarded, suggesting a scale-dependent, hierarchical distribution of transport resistances (“constrictions” in the channels) and of shortcuts (connecting “bridges”) between adjacent channels. The experimental evidence from these studies is confirmed by molecular dynamics (MD) simulation in the range of atomistic displacements and rationalized with a simple model of statistically distributed “constrictions” and “bridges” for displacements in the micrometer range via dynamic Monte Carlo (DMC) simulation. Both ranges are demonstrated to be mutually transferrable by DMC simulations based on the pore space topology determined by electron tomography.

Porous solids and in particular porous semiconductors in which the transport properties are enhanced by the introduction of pore networks have found widespread application in several modern technologies^1^,^2^. As a part of this strategy, the addition of spatial anisotropy within the pore network may provide the highest rates of matter transport and thus the highest production rates. However, attaining ideal anisotropy over macroscopic distances is an extremely challenging task, which has attracted increasing interest, as shown by the rapidly increasing number of publications dealing with synthesis of highly anisotropic pore structures^3^,^4^,^5^,^6^. At the same time, methods of structural and transport characterization for such materials are rather limited. In contrast to zeolitic materials, these materials generally do not have the well-ordered pore networks required for rigorous theoretical analysis. Establishing the relationships between transport and structural anisotropy in this case is a non-trivial task.

With obtainable pore sizes ranging from several nanometers to hundreds of nanometers, nanoporous silicon (pSi)^7^ is unique since its properties, including the mass transfer rates within the pores, can be tuned by varying the pore size. That is why pSi has found widespread use in many different applications ranging from solar cells^8^ to cancer therapy^9^,^10^,^11^, protein separation^12^, biosensing^13^ and design of contrasting agents for magnetic resonance imaging^14^. The coupling of light propagation and capillary condensation in pSi, thus allowing the light-assisted manipulation of the mass transfer properties, provides a particularly prominent example^15^.

While macroporous silicon is known to contain non-intersecting smooth channels^16^,^17^, the melting-freezing and evaporation-condensation behavior of guest molecules in small-pore pSi (≤20 nm) suggests a more complex pore structure^18^,^19^,^20^,^21^,^22^. The occurrence of the intersections between adjacent channels was examined by setting up experiments sensitive to molecular transport in the pore spaces of pSi. Thus, the kinetics of the photochemical alkylation of hydrogen-terminated pSi were probed and analyzed using a diffusion model allowing for diffusion anisotropy of the reactant species^23^. In these studies, the resulting data could only be explained by assuming a negligible diffusion rate between different channels. In contrast, diffusion studies using pulsed field gradient NMR revealed pronounced diffusion anisotropy^24^. In order to resolve these issues we have carried out a systematic study of the transport properties of pSi over a broad range of length scales. Combining the evidence from diffusion measurements and simulations, diffusion anisotropy in pSi has now been subjected to an in-depth analysis covering the entire range from elementary steps up to macroscopic translational displacements of guest species in a porous material with tunable pore space anisotropy.

## Results and Discussion

**Figure 1:**
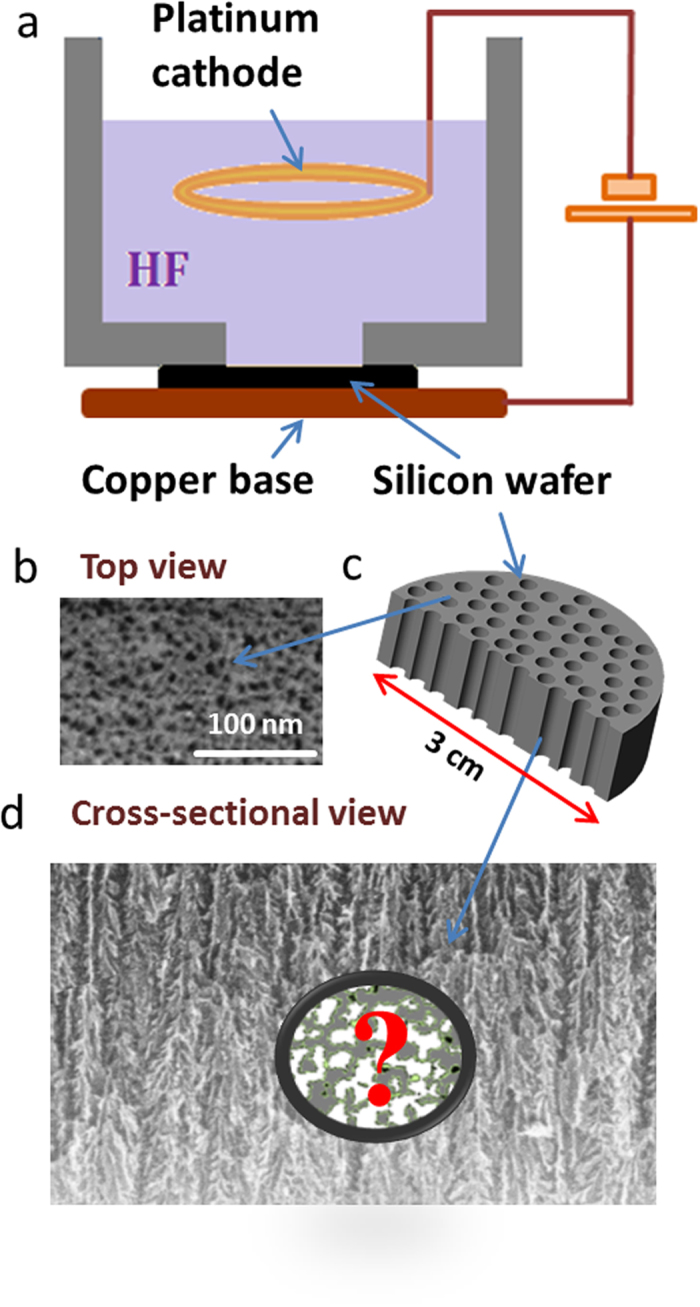
(**a**) Surface etching by hydrofluoric acid in combination with electric current applied to doped silicon wafers generates a system of channel pores. (**b**) Electron microscopy images of the pore entrances. (**c**) Idealized view with indicated pSi film extension, the film thickness is 100 μm. (**d**) A cross-sectional view of the pore network.

Figure 1a illustrates the fabrication procedure of the host material used in our studies. It is based on the etching of purposefully doped silicon wafers in combination with the application of electrical current and referred to as electrochemical anodization^25^. The process is known to lead to channel formation in the direction governed by the atomic structure of the substrate and its orientation. In our case, the pore growth direction was perpendicular to the plane of the wafer as indicated schematically in Fig. 1c.

**Figure 2:**
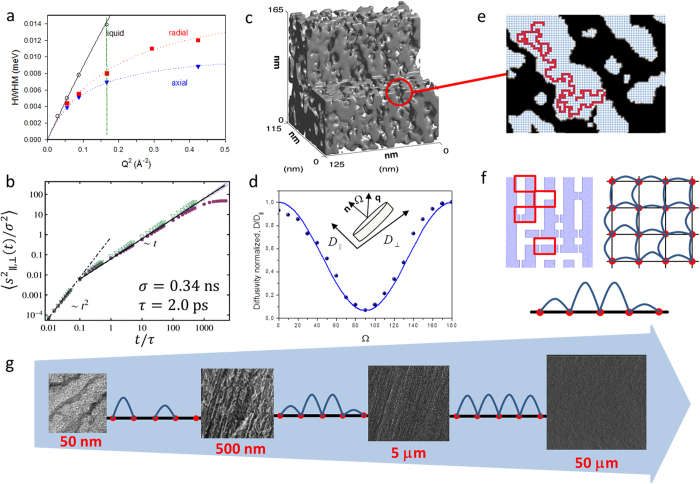
(**a**) Primary data of Quasi-Elastic Neutron Scattering (half-width at half-maximum (HWHM) of energy distribution). The vertical green line indicates the lower limit in the scattering vectors (and, correspondingly, the upper limit in the displacements) considered in a previous study^42^. (**b**) Mean square displacements in molecular dynamics simulations in free liquid (triangles), and parallel (open circles) and perpendicular (filled circles) to main channel direction. (**c**) The topological information provided by 3d electron tomography. (**d**) Orientation-dependent diffusivities evidenced by PFG NMR measurements performed by varying the direction of transport observation, i.e. by orientating pSi film with respect to the direction of the magnetic field gradient along which the molecular displacements are probed^53^. The solid line is the best fit of Eq. (7) to the experimental data. (**e**) Scheme of the simulation matrix used for short-range displacements based on the topology of (**c**). (**f**) Simplified simulation network (only 2d cross-section is shown) with randomly distributed “bridges” and “constrictions”. The squares indicated on top left panel show the four variants in which the unit cells of the simulation network may occur, namely accommodating a straight channel segment only (top), a channel segment with a connection (a “bridge” to an adjacent channel, centre right), a channel segment with “constriction” (centre left) and both “bridge” and “constriction” (bottom). From the theoretical perspective, the model is analogue to random walks between the lattice nodes (red dots), with spatially-distributed hopping times (or respective transport resistances as schematically shown by the different arch heights (in blue) in the top right figure). The lower panel indicates an integrated 1d profile for diffusion perpendicular to the channel direction. (**g**) Visualizing displacement-dependent diffusivities: increasing displacements are accompanied with an increase in the maximum values of transport resistances, in parallel with a decrease in their distribution widths as indicated by changing the arch-height distributions (increase of the length scale is analogue of coarse-graining of the lattice in (**f**), leading to averaging of the hopping times).

While channel openings in the wafer surface consistent with this simple view are seen to appear in the electron micro-images (Fig. 1b), experimental evidence on pore architecture in the wafer interior suggests a more complex structure, as exemplified in Figs 1d and 2g by electron micrographs and in Fig. 2c by a 3d-pore space representation acquired by electron tomography. Both types of microscopic imaging reveal clear deviations from a regular arrangement of parallel, non-intersecting channels as suggested by the appearance of channel openings clearly separated from each other on the external surface (Fig. 1b) and, moreover, also well-known from macroporous silicon^16^. Given the experimental evidence of mutual connectivity, adsorption phenomena in this type of material should not be analyzed on the basis of the commonly used assumption of “mutual independence” of adjacent pore channels^19^,^24^,^26^.

These deviations may be expected to have a significant effect on mass transfer within these materials and thus on their technological performance^7^,^12^,^15^,^22^,^27^,^28^,^29^. Deviations of particular relevance for mass transfer include connections between adjacent channels, which will enable macroscopic displacements of guest molecules in the transverse direction, i.e. perpendicular to the main channel direction^24^. On the other hand constrictions along the channels may be expected to reduce the rate of mass transfer in the channel direction. Both these features are combined within the scheme shown on the left of Fig. 2f.

For the exploration of mass transfer over macroscopic dimensions in both axial and transversal directions (i.e. in the direction of the channel axes and perpendicular to the channels) we have exploited the pulsed field gradient (PFG) technique of NMR^30^,^31^. Being able to record molecular displacements from a few hundreds of nanometers up to hundreds of micrometers, this technique is ideally suited for in-depth studies of molecular translational dynamics in such systems^32^. As an example of the outcome of the orientation-dependent PFG NMR diffusion studies, Fig. 2d shows the variation of the diffusivity as obtained using PFG NMR for a fixed observation time with the angle between the observation direction (direction of the magnetic field inhomogeneity, i.e. the magnetic field gradient) and the main channel direction (perpendicular to the wafer plane). We note that, with the sample under study, the diffusion flux perpendicular to the main channel direction is reduced by about one order of magnitude in comparison with the flux in channel direction. The literature already contains many reports on the observation of diffusion anisotropy in porous materials^33^, including mesoporous solids like SBA-15^34^. However, the critical point revealed by the present study is that the anisotropy factor is scale-dependent and this dependency has been followed over several orders of magnitude.

The ability to make direction-dependent diffusion measurements over varying time and space scales makes PFG NMR a most sensitive tool for the detection and exploration of transport phenomena deviating from the common pattern of “normal diffusion”


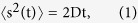


where the mean square displacement 〈s^2^(t)〉 in a certain direction scales in proportion with the observation time t, with the factor of proportionality D referred to as the self-diffusivity^30^,^31^. Prominent examples of the application of PFG NMR for probing anomalous diffusion include the reptation of polymer chains^35^, single-file diffusion in zeolites^36^ as well as the transition from intra- to intercrystalline diffusion^37^. In such cases it is common practice to use Eq. (1) for the definition of a scale-dependent diffusivity


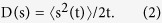


**Figure 3:**
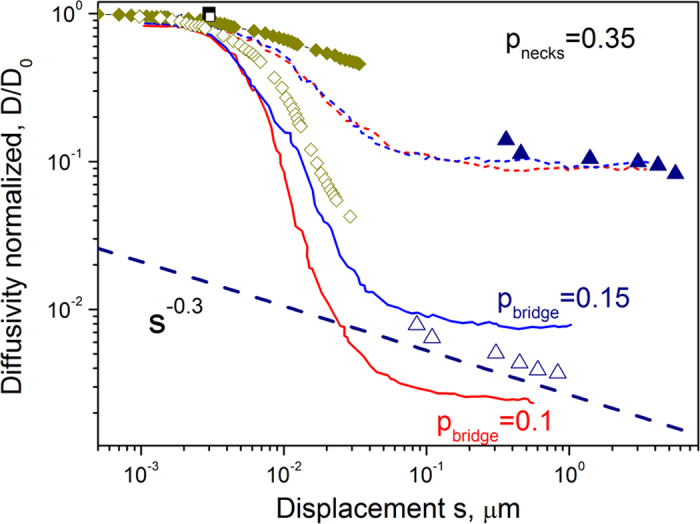
PFG NMR diffusivities at room temperature (20 °C) with TEHOS in transversal (open triangles) and axial (full triangles) directions. The QENS diffusivities (cyclohexane, 20 °C) in axial and transversal directions (full and open black squares) approach, essentially, that in the free liquid (see Fig. 2a). The symbol sizes in the figure are representatives of the experimental error for the diffusivities measured. Dynamic Monte Carlo simulation results determined by using the pore space model shown in Fig. 2f (left) are represented by dotted and continuous lines for the diffusivities in axial and transversal directions, respectively, those obtained with the pore space model as resulting from electron tomography (Fig. 2e) by diamonds, where filled and open symbols refer to the diffusivities in axial and transversal directions. The broken line shows the scale dependence of the diffusivity in a system with hierarchically organized transport resistances (Fig. 2f (right) and 2 g) giving rise to a time exponent κ = 0.87 in Eqs (3) and (4).

A summary of the PFG NMR diffusivities obtained in the present studies with the pSi introduced in Fig. 1 as a host system is included in Fig. 3, which we have prepared in order to summarize the outcome of all the experimental and computational techniques applied in the present study. All diffusivity data (D(s)) are presented as relative units, normalized to their values in free space (D_0_). The PFG NMR diffusivity data in Fig. 3 have been measured using TEHOS (=tetrakis-2-ethylhexoxy-silane, Aldrich, Germany). Its relatively low bulk-phase diffusivity (*D*_0_ = 7.8 × 10^−11^ m^2^/s at 20 °C) makes it a sensitive guest molecule for probing pore spaces over short distances.

The impact of both types of structural peculiarities as illustrated in Fig. 2f (left) is immediately recognized in the representation of the PFG NMR diffusivities in Fig. 3. There is a clear reduction of the diffusivities in comparison with the free liquid even in the main channel direction which may be taken as a consequence of the channel disorder (modelled here by constrictions) as revealed by the micrographs. Simultaneously, even if further slowed down by another order of magnitude, diffusion is also seen to occur (over macroscopic distances) in the directions perpendicular to the main channels. This implies the existence of connections between different channels.

The simulation data included in Fig. 3 show the extent to which the experimental diffusivities are reproduced by the results of the modelling studies. Perhaps surprisingly, even the simplest assumption of only one type of channel constriction (modelled by the inclusion of narrow necks of density p_necks_ = 0.35 corresponding to a mean distance of about 6 nm/0.35 = 17 nm between adjacent constrictions) and of channel interconnection (of density p_bridges_ indicated in Fig. 2f (left)) is seen to provide simulation data in good agreement with the main details of the experimental results, yielding coinciding trends in their dependence on the relevant displacements. The diffusivities simulated in either direction are seen to approach, for sufficiently large displacements, a constant value. This is in complete agreement with the model which implies uniformity when the displacements are sufficiently large in comparison with the characteristic disorder length scales in both directions. Such behavior might – within the limits of accuracy as represented by the scattering of the experimental points – also be attributed to the results of diffusion measurement in the channel direction.

There is, however, a clear tendency that the diffusivities perpendicular to the main channel direction continue to decrease with increasing displacements. This is also nicely corroborated by the simulation data obtained following the scheme shown in Fig. 2f, left (continuous lines in Fig. 3): While for short displacements (∼100 nm) there is a reasonably good fit to the experimental data with the higher probability of encountering connecting bridges (p_bridges_ = 0.15, corresponding to mutual separations of about 40 nm), for the largest displacements better fits are seen to require a decreasing probability of the occurrence of connecting bridges (towards p_bridges_ = 0.10, corresponding to mutual separations of about 100 nm). This is the well-known behavior of hierarchically organized materials where increasing displacements have, as a rule, to compete with increasing confinement^38^. In the given case this would mean that the different channels are not uniformly interconnected. Evidently the propagation rate from one channel to an adjacent one decreases with increasing distance from the starting point.

Such a situation is automatically ensured in the presence of (i) spatial hierarchies, such as for spatially-correlated distributions of the transport resistivities^31^,^39^, or (ii) temporal hierarchies, such as for distributions of the waiting times^40^. To capture the temporal pattern obtained for the system under study, a concerted action of both these mechanisms would be required: (i) the mesoscopic structural heterogeneities in porous silicon, giving rise to a correlated distribution of p_bridges_ over the length scale from several tens of nm to several μm, and (ii) a distribution of the separation between two connections between two channels, giving rise to a distribution of the inter-channel hopping times. This situation is illustrated in Fig. 2f and g with the blue loops. The height of each loop (“arch”) stands – in terms of the “resistance” which the diffusing molecules have to overcome – for the mean passage time from one channel to an adjacent one (as symbolized by the red dots). With Fig. 2g the distribution of these resistances appears to decrease with increasing distances. Under these conditions, deviating from Eq. (1), the mean square displacement may be shown to increase less than linearly with time, following the expression


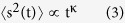


with a time exponent κ < 1. Combining equation (3) with the definition of a scale-dependent diffusivity as provided by Eq. (2) yields


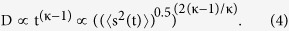


The broken line in Fig. 3 reflects the scale dependence of the PFG NMR diffusivities perpendicular to the main channel direction, yielding a value of 2 (κ−1)/κ ≈ −0.3. From this relation, the time exponent of the associated mean square displacements is κ = 0.87 rather than 1.

The diffusivities accessible by Quasi-Elastic Neutron Scattering (QENS)^31^,^41^ are based on measurements covering molecular displacements over nanometers and, hence, over a space scale dramatically different from that of PFG NMR. The QENS diffusion data are thus positioned in the representation of diffusivities in Fig. 3 in the very beginning of the considered space dependence. We note that now, within the limits of accuracy, the diffusivities in the axial and transversal directions coincide with each other and, moreover, are close to the diffusivity in the free liquid. This is evidenced by the slopes of the experimental data approaching one another at small wave-vector transfers *Q*. Molecular displacements as covered during the measurement are thus found to be small enough so that they remain essentially unaffected by the existence of the confining walls. Moreover, also any substantial “long-range” influence of the walls on impeding the molecular mobility appears to be below the limit of measurability. Given some deviations of these findings from literature reports (namely a slight slowing down of the diffusivity in comparison with the free liquid in ref. 42 and even diffusivity enhancement in the transversal direction, compared with axial diffusion^43^), the situation as considered in our QENS measurements has been taken as a starting point for molecular dynamic simulations. The QENS diffusivities were found to be reproduced by the MD simulations in an essentially perfect way, indicating that within the uncertainty of the measurements (~10%) with the given system diffusivities in axial and transversal directions for displacements large compared to the elementary steps (0.34 nm) and small to the channel diameter (6 nm) should be expected to coincide. The situation will, as a matter of course, change with decreasing pore size where, as well known with the behavior of colloidal particles in channels^44^, diffusion rates in the transverse direction will eventually fall below axial diffusion.

Electron tomography^45^,^46^ allows simulations of guest diffusivity with a much more precise involvement of the structural details than with the oversimplified model assumptions of Fig. 2f (left). The potential of this technique is illustrated by Fig. 2c, revealing that pore space polymorphism is found to dramatically exceed that of the simple model considered in Fig. 2f. It has to be emphasized, however, that the DMC simulations performed using the model pf Fig. 2f, resulting in the continuous and broken lines in Fig. 3, reproduce the main properties of the pore space in Fig. 2c. In particular, the space scale derived in these simulations for the separation between adjacent “constrictions” and “bridges” is seen to reflect a reasonable order of magnitude.

The simulations with the pore space model reconstructed from electron tomography data can clearly only be performed on the basis of the structural information provided by a very small number of particles with extensions from a few up to a few hundred nanometers (see, e.g., Fig. 2c) which have more or less incidentally been broken out of pSi discs of typically 100 μm thickness and extensions of cms (Fig. 1c). It is therefore difficult to define precisely the extent to which the structural information thus provided really corresponds with the actual situation as traced in the PFG NMR experiments.

With these limitations in mind, the results of the dynamic Monte Carlo simulations with the reconstructed pore space (Fig. 2e) as shown by the diamonds in Fig. 3 may be considered to match reasonably well with the experimental diffusivity data of the PFG NMR measurements, filling the gap between the ranges of measurement so far accessible between QENS and PFG NMR. This is in particular true for the diffusivities in the transversal direction where an extrapolation of the simulation results leads to the measured points. In searching to explain why a similar procedure provides a less good fit of the diffusivities in axial direction, one has to consider the possibility that the structure of the monitored pieces may not be really representative of the sample under investigation in the PFG NMR diffusion studies. There is, however, also the possibility to generate exactly the observed dependence by assuming that there are significant transport resistances (with a mutual distance between the largest displacements in main channel direction considered in the simulations (~40 nm) and the shortest distances of the measurement (~400 nm). In the context of our model this would imply particularly pronounced constrictions. In such a situation the diffusivities as attainable by PFG NMR are known to drop from higher to lower values for displacements increasing from below the spacing between these resistances towards the range beyond^47^.

## Conclusions

Microscopic techniques of measurement together with structure-oriented techniques of simulation have been applied for an in-depth study of guest diffusion in nanoporous silicon. Displacements from molecular distances up to micrometers were recorded. Most importantly, in addition to the main flux through the channels, guest diffusion was found to occur also in the directions perpendicular to the main channels, suggesting the existence of “bridges” between adjacent channels. Simultaneously, diffusion in the direction of the main channels was found to be notably reduced in comparison with the short-range diffusivities, suggesting that molecular propagation in the main channels is probably impeded by the existence of “constrictions”. The experimental diffusivity data are shown to be nicely corroborated by the results of molecular simulations based on the structural information provided by pore space tomography. In this way both the absolute values of the diffusivities in the “axial” and “transverse” directions and their ratio, i.e. the extent of diffusion anisotropy, become a function of the relevant space scales. The scale-dependent diffusivities in turn contain valuable information about the real structure of nanoporous silicon which is not easily accessible by other means.

## Methods

### Samples

Porous silicon was prepared by electrochemical etching of highly B-doped *p*-type silicon wafers with (100) crystallographic orientation and with a resistivity of 2–5 mΩcm. Anodization was performed using a home-built wet-etching cell with horizontal orientation of the silicon wafers. The lower electrical contact to the wafers was provided via a copper base. A cylindrical cell containing the electrolyte was placed on the top of the silicon wafer. The diameter of the porous silicon layer formed on the wafer surface was controlled using an O-ring with an inner diameter of 15 mm. A platinum wire with a diameter of 1 mm immersed in the electrolyte was used as the electrode. Etching was performed at dark conditions and at room temperatures. The electrolyte contained 48% aqueous HF solution mixed in equal volumes with ethanol. The anodization current density *j* was 20 mA/cm^2^. The porous films were formed to have a thickness of about 100 μm. Upon formation they were detached from the substrate pores by applying a polishing step. The average pore size of about 6 nm and 7.5 nm in the porous silicon was determined using NMR cryoporometry and gas adsorption methods.

### Field-emission scanning electron microscopy

These studies were made in secondary electron contrast at an acceleration voltage of 2 kV employing a JEOL JSM-6700F microscope. Observations were made on the surface of as-received specimen or broken cross-section without applying any surface coating.

### Electron tomography

Electron tomography was performed on a Tecnai 20 (FEI) transmission electron microscope operated at 200 kV in bright field imaging mode. A series of tilt images were acquired using a bottom mounted TVIPS CCD camera at nominal magnification of 100,000 over an angular range of ±74° at increments of 2°. Alignment of the acquired tilt series and subsequent reconstruction using WBP (weighted back projection) algorithm were performed with IMOD software package^48^. Resulting reconstruction was binned by 2 and had a voxel size of (0.46 nm^3^).

### QENS

The experiments were performed using the IN5 spectrometer, at the Institut Laue-Langevin. The incident neutron wavelength was of 8 Å. Neutrons scattered by the sample were analyzed at different angles, corresponding to different wave-vector transfers, Q, ranging from 0.16 to 1.3 Å^−1^. The elastic energy resolution, measured with a vanadium standard, was fitted by a Gaussian function, with a half-width at half-maximum (HWHM) varying from 11 μeV at small Q to 13 μeV at large Q. Two samples were prepared after desorption at 200 °C of two porous silicon films: the first one was saturated on a vacuum line with cyclohexane and the second one was left empty. The samples were transferred inside a glovebox into slab-shaped aluminum cells. The scattering from the empty wafer was subtracted from the spectra recorded with the loaded sample. In order to study diffusion anisotropy, Q has to be oriented with respect to the main channel axis. For this purpose, it is usual to do measurements at two angles, 45°–135°, between the incident beam and the sample (here the wafer plane), in order to probe motions parallel and perpendicular to the main channel direction. However, this is only correct close to a scattering angle of 90°. We have performed additional measurements for other angles of the wafer plane: 30°–120°, and 15°–105°, to determine more accurately axial and transversal diffusion at lower Qs. All data were recorded at 300 K, so that the contribution from a fraction of immobile molecules (as in ref. 42 in the case of n-hexane) was not needed.

The spectra were fitted by a rotational diffusion model convoluted by a translational motion. The HWHM of the translational component is plotted in Fig. 2a as a function of Q^2^. For Fickian diffusion, a linear variation of the HWHM is expected. Deviations from a straight line are a signature of jump diffusion. Spectra measured in the Q range 0.16–0.65 Å^−1^ could be fitted simultaneously using a jump diffusion model with a distribution of jump lengths (the solid lines in Fig. 2a). Differences are observed at large Q values, indicating different parameters for the elementary jumps (such as jump rate), but the main result is that the slopes at low Q are approaching one another, which means that the different diffusivities probed over a length scale of 4 nm are very close. The different experimental conditions used in ref. 42 (the vertical line in Fig. 2a) explain why a slightly lower diffusion was derived for n-hexane confined in a porous silicon matrix compared to the bulk liquid^42^.

### Pulsed field gradient ^1^H NMR diffusometry

The experiments were performed using a 400 MHz NMR spectrometer equipped with a home-built pulsed field gradient unit producing the extremely-large gradient of g = 35 T/m with very short pulse rise and fall times. This allowed to probe a broad range of the translational displacements of the molecular species from about 70 nm to about 1 μm. All diffusion experiments were performed using the 13-interval pulse sequence. The spin-echo signals S, attenuated due to diffusion in the presence of the inhomogeneous magnetic field, were measured by incrementing the linear gradient intensity and by keeping all other parameters of the pulse sequence constant. In this case, the signal S is found to be


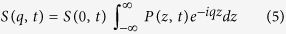


where q = γδg is the wave-number, γ( = 2.8 × 10^8^ rad/Ts) is the gyromagnetic ratio for protons, δ is the duration of the gradient pulses, and t is the diffusion time which is controlled in the experiments. In Eq. (5), P(z, t) is the ensemble-averaged probability density that during the observation time t the molecules will be displaced by z in the direction of the applied magnetic field gradient. In the low q limit, i.e. by accounting only the leading, non-disappearing term in q, one finds^30^


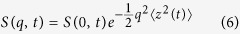


Notably, Eq. (6) is valid for assessing the mean square displacements quite generally, irrespective of the underlying diffusion laws. In the particular case of normal diffusion, 〈z^2^(t)〉 = 2Dt, and the diffusivities can readily be obtained from the slope of ln(S(q, t)) plotted vs. q^2^t. The same procedure is applicable in the case of anomalous diffusion, but, in this case, D becomes a function of the observation time^37^,^49^,^50^,^51^,^52^.

For the diffusion measurements, tetrakis(2-ethylhexoxy)silane (TEHOS) was used as a probe liquid. TEHOS molecules have a roughly spherical shape, with a diameter of about 1 nm, and possess a low bulk diffusivity of 7.8 × 10^−11^ m^2^/s at T = 20 °C. The porous silicon films were evacuated under 150 °C for several hours and TEHOS was added. After the entire mesopore volume was saturated by TEHOS due to capillary action, the external surface was cleaned to remove excess TEHOS. To prove the diffusion anisotropy, the pSi films were orientated in a special NMR holder under a certain angle Ω with respect to the magnetic field gradient (typically z-axis) and the apparent diffusivities D(Ω) were measured as a function of Ω (see Fig. 2d)^53^. The expected pattern


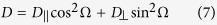


was found to agree perfectly with the experimental data. For the data of Fig. 3, only measurements with Ω = 0 and Ω = 90° were considered.

### NMR cryoporometry

The method of NMR cryoporometry^54^,^55^ exploits the occurrence of the melting temperature shift for fluids confined in mesoporous solids for their structural characterization. In our approach, we first cooled the pSi sample saturated with water down to −35 °C such that all water froze. Thereafter, upon warming the sample, the NMR Hahn-echo signal intensity was measured^20^,^56^,^57^ as shown in Figure S1 of Supplementary Information. The echo-formation time of 6 ms was chosen to completely suppress any contribution to the echo signal from the frozen water phase due to notably shorter nuclear magnetic relaxation times in ice (shorter than 100 μs). The thus measured curve (see Supplementary Information) represented the relative fraction of liquid water. By using the Gibbs-Thomson law T_0_ − T = K/d to correlate the suppression temperature and the pore size, the pore size distribution function was derived (Figure S2 of Supplementary Information). In this equation, K = 56 Knm is a constant determined by the thermodynamic parameters of water, T_0_ is the bulk transition temperature, and d is the pore diameter. It is known that the pore sizes determined in geometrically disordered mesoporous solids obtained with this method from the melting transition are biased towards smaller pore sizes^58^,^59^. The average pore size delivered by NMR cryoporometry is therefore slightly smaller than that obtained using gas adsorption.

### Gas sorption

Nitrogen adsorption experiments have been performed at 77 K using Autosorb-1-MP (Quantachrome Corporation, Boynton Beach, FL), which is dedicated to the standard characterization of nanostructured materials. The obtained hysteresis loop (see Figure S3 of Supplementary Information) is typical of disordered mesoporous materials, and of mesoporous silicon in particular^24^,^26^, and the isotherm is classified as type IV according to the IUPAC classification^60^. The pore size distributions shown in Figure S3 of Supplementary Information were obtained with the advanced state-of-the-art nonlocal density functional theory (NLDFT)^61^. For the consistency check, additional measurements using argon adsorption were performed. By showing the coincidence of the pore size distributions obtained using argon and nitrogen, any artefacts related to the occurrence of disturbing cavitation effect could be excluded. However, the differences between the widths of the pore size distribution curves obtained from nitrogen and argon adsorption and desorption branches, respectively, clearly indicate the presence of the pore blocking effects caused by the the presence of constrictions of sizes larger than ca. 5-6 nm, which is in line with the findings from the diffusion experiments.

### Molecular Dynamics Simulation

We perform molecular dynamics simulation of a simple fluid in a cylindrical nanochannel (see Figure S4 of Supplementary Information). The solvent was modelled as spherical particles of size σ, and mass m, interacting via the Lennard-Jones potential U(r) = 4ϵ[(σ/r)^12^ − (σ/r)^6^] truncated at r_c_ = 2.5σ. The walls of the cylindrical channel were explicitly modelled from a cylindrical cut of the initial FCC lattice. The atoms in the cylindrical channel were bound together by the FENE potential U(r) = −0.5 κR_0_^2^ log[1 − (r/R_0_)^2^] with κ = 30 ϵ/σ^2^ and R_0_ = 1.5 σ. As usual, lengths, times and energies were measured in units of σ, τ≡√((mσ^2^)/ϵ) and ϵ, respectively. For liquid Argon, the values correspond to σ = 0.34 nm, ϵ/k_B_ = 119.8 K, m = 0.03994 kg/mol and τ = 2 ps. In the simulations, the total number of particles was 32896, of which 11936 particles made up the solvent and the rest of the particles form the wall of the cylindrical channel. The length of the simulation box along the z-direction was chosen to be 44.8 σ, and along the x and y direction was 25.2 σ, with periodic boundary condition imposed only along z-direction. The radius of the nanochannel was taken to be R = 10 σ ≈ 3 nm. The mean density of the solvent within the nanochannel was 0.823 σ^−3^. In a typical simulation, the system was first equilibrated using a Nosé-Hoover thermostat^62^ at a prescribed temperature of 0.75 ϵ/k_B_ with an integration time step of δt = 0.005 τ. After the initial equilibration process, the coordinates and momenta of the fluid particles were measured and the mean-square displacement along and perpendicular to the channel were calculated from the time trajectories. The mean-square displacement was further averaged over four independent simulation runs of length 5000 τ, corresponding to a physical duration of 10 ns.

The measured mean-square displacement (MSD) along the channel direction (z-direction) exhibits a ballistic behavior at short time scales with 〈Δr^2^〉 = (3k_B_T/m)t^2^ and crosses over to a diffusive behavior with 〈Δr^2^〉 = 2Dt as shown in Fig. 2b. For displacements smaller than the radius of the cylindrical channel, the mean-square displacements along and perpendicular to the channel exhibit the linear behavior. However, at very late times, when the displacements are comparable to the channel width, the mean-square displacement saturates to a value R^2^, where R^2^ is the maximum accessible transversal distance.

### Dynamic Monte Carlo simulations

The simulations were based on a lattice model as shown in Fig. 2f. The basic elements of the lattice were collections of parallel, rectangular channels with an edge length of 6 sites. One site length was chosen to be 1 nm to resemble the diameter of the TEHOS molecules. This choice did, simultaneously, correspond with the mean channel diameter as determined by NMR cryoporometry^57^,^58^ and by nitrogen and argon gas adsorption^60^. The separation between the channels was 6 sites. Along the channel main axes constrictions (“necks”), namely rectangular channels of one-site width and of 3 sites length, of density (probability per unit channel length) p_necks_ were distributed. These “necks” may be considered, in some way, to assume the role of diffusion barriers which in nanoporous materials are known to occur both in the intracrystalline space^47^ and on the external surface^63^,^64^. Each unit cell was 6 sites long. Connecting bridges to one of the four neighbor channels occurred with the probability p_bridges_ per unit channel length. The orientation of the bridge was chosen randomly from 4 possible directions. Diffusion was modeled as a site-to-site random hop process in 6 possible directions. If a site was occupied by the pore walls (grey fields in Fig. 2f left), the jump was forbidden and the particle remained in the initial site. The hopping time was selected to resemble the bulk diffusivity of TEHOS. The diffusivities were traced along and perpendicular to the channel directions. Selected results with different p_necks_ and p_bridges_ are shown in Figures S6 and S7 of Supplementary Information. Note that, with the site length chosen to be 1 nm, the Dynamic Monte Carlo simulations did not really attain the range of totally unrestricted diffusion in the limit of vanishing observation times, given the appreciably large number of sites adjacent to the boundary. It is due to this reason that in Fig. 3 the Monte Carlo simulation data are slightly below the diffusivities resulting in both QENS and MD simulation.

Diffusion perpendicular to the channels may be seen as a 2d diffusion process with disordered transition rates^40^. Indeed, in order for a particle to approach a neighbor channel it must travel in the axial direction before it finds a bridge in the required direction. The net effect of the diffusive axial displacements is thus to determine the distribution *ψ(τ*) of the transition or waiting times *τ*. With the given *p*_bridges_, the distribution *φ(l*) of the separations *l* between two bridges between two neighbor channels is 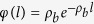, where 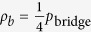. The average time *τ* to reach a bridge scales as *τ* ∝ *l*^2^/*D*_||_, hence 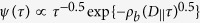. Because this distribution does not provide heavy tails, alone it cannot yield anomalous diffusion in the whole range of the displacements and ensures the formation of the plateau as seen in Fig. 3.

## Additional Information

**How to cite this article**: Kondrashova, D. *et al*. Scale-dependent diffusion anisotropy in nanoporous silicon. *Sci. Rep.* **7**, 40207; doi: 10.1038/srep40207 (2017).

**Publisher's note:** Springer Nature remains neutral with regard to jurisdictional claims in published maps and institutional affiliations.

## Supplementary Material

Supplementary Graphs
