# Supplementary material for: Scale-dependent diffusion anisotropy in nanoporous silicon
Source: Sci Rep. 2017 Jan 20;7:40207. doi: 10.1038/srep40207 (PMC5247748; doi:10.1038/srep40207)
Supplement: Supplementary Graphs [file srep40207-s1.pdf]

Supplemenary Information for **Scientific Reports**

Scale-dependent diffusion anisotropy in nanoporous materials

Daria Kondrashova[1,2]; Alexander Lauerer[1], Dirk Mehlhorn[1], Hervé Jobic[3]; Armin Feldhoff[4], Matthias Thommes[5], Dipanjan Chakraborty[6], Cedric Gommes[7], Jovana Zecevic[7], Petra de Jongh[7], Armin Bunde[2], Jörg Kärger[1]*, Rustem Valiullin[1]

[1]University of Leipzig, Faculty of Physics and Earth Sciences, Linnéstraße 5, D-04103 Leipzig, Germany. [2]University of Gießen, Institute of Theoretical Physics, Heinrich-Buff-Ring 16, D-35392 Gießen, Germany. [3]Institut de Recherches sur la Catalyse - CNRS 2, Avenue Albert-Einstein, F-69626 Villeurbanne Cedex, France. [4]Leibniz University Hannover, Institute of Physical Chemistry and Electrochemistry, Callinstr. 3-3A, D-30167 Hannover. [5]Quantachrome Ins., 1900 Corporate Drive, Boynton Beach, Florida 33426, USA.[6]Indian Institute of Science Education & Research, Sec 81, SAS Nagar, Manauli PO, Punjab, India.[7]Utrecht University, Department of Inorganic Chemistry and Catalysis, Sorbonnelaan 16, NL-3584 CA Utrecht

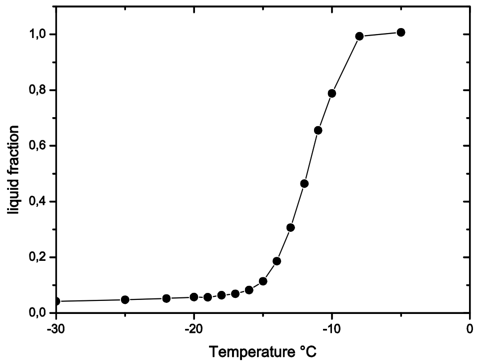

Figure S1. Fraction of liquid water in pSi upon warming.

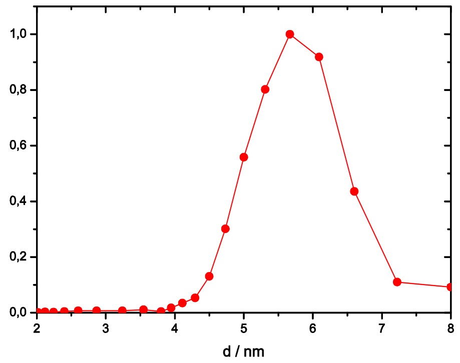

Figure S2. Pore size distribution in pSi as obtained from the melting curve shown in Fig. S1.

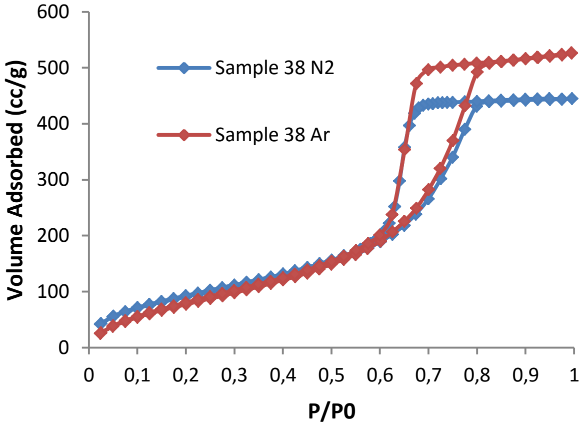

N2 pore size distributions

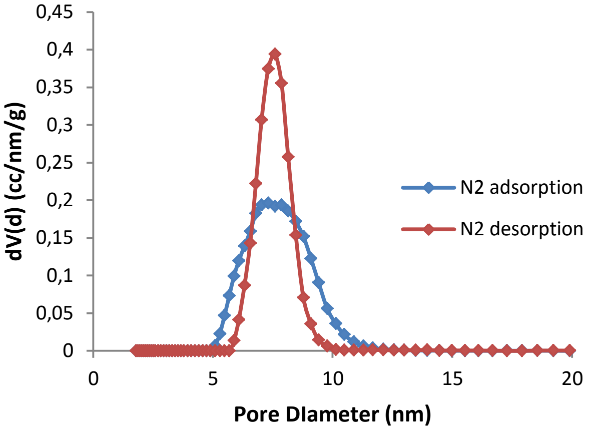

Ar pore size distributions

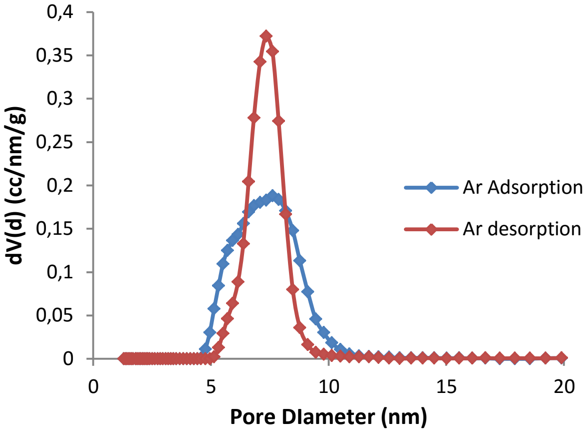

Pore size distributions from desorption kernel

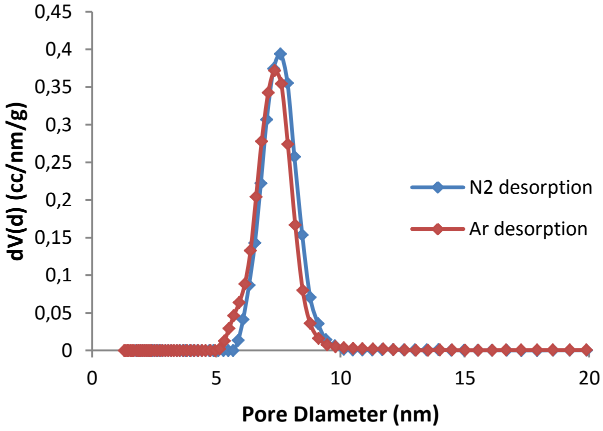

Pore size distributions from adsorption kernel

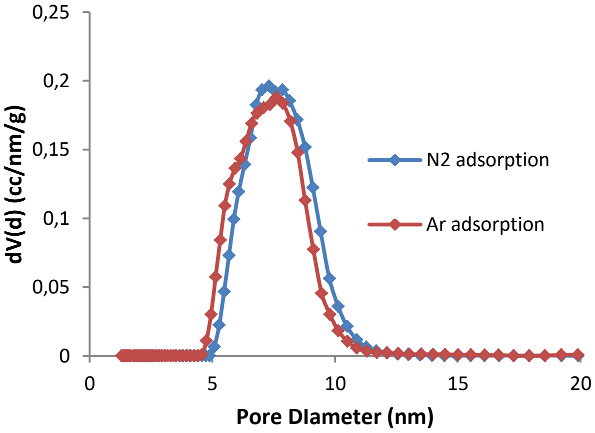

Figure S3. Nitrogen and argon adsorption isotherms and respective pore size distributions derived using NLDFT.

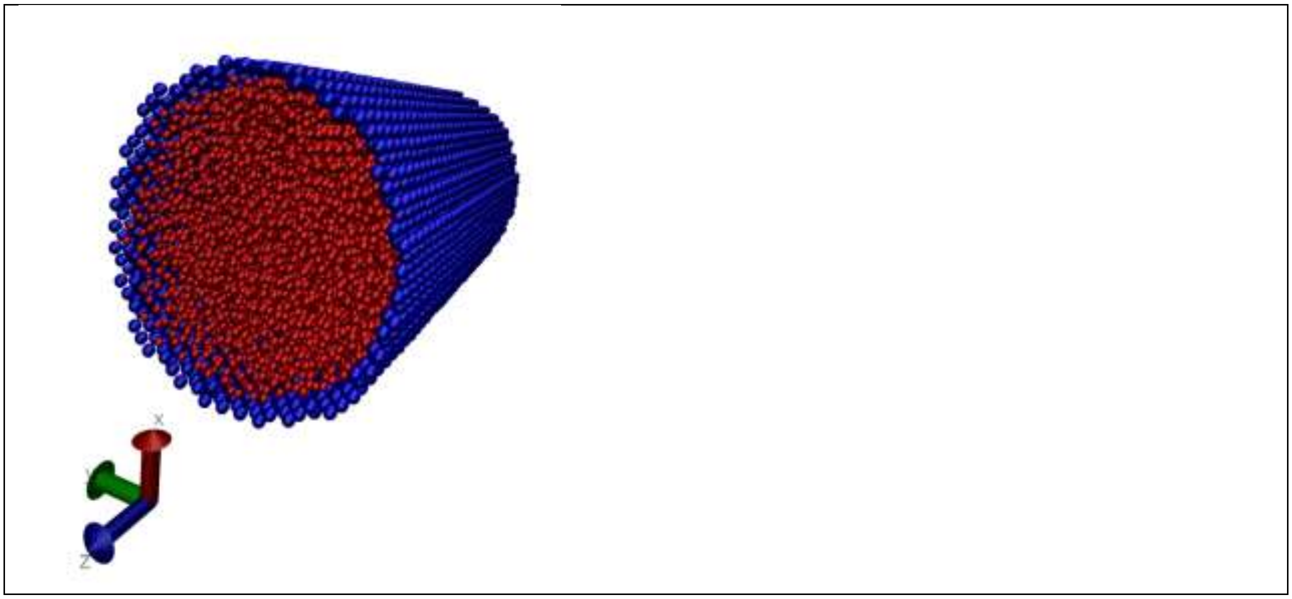

Figure S4: A snapshot of the molecular dynamics simulation depicting the solvent (red colored) moving inside a nanochannel of approximate diameter of 3 nm.The channel is along the z-direction as shown by the arrow heads.

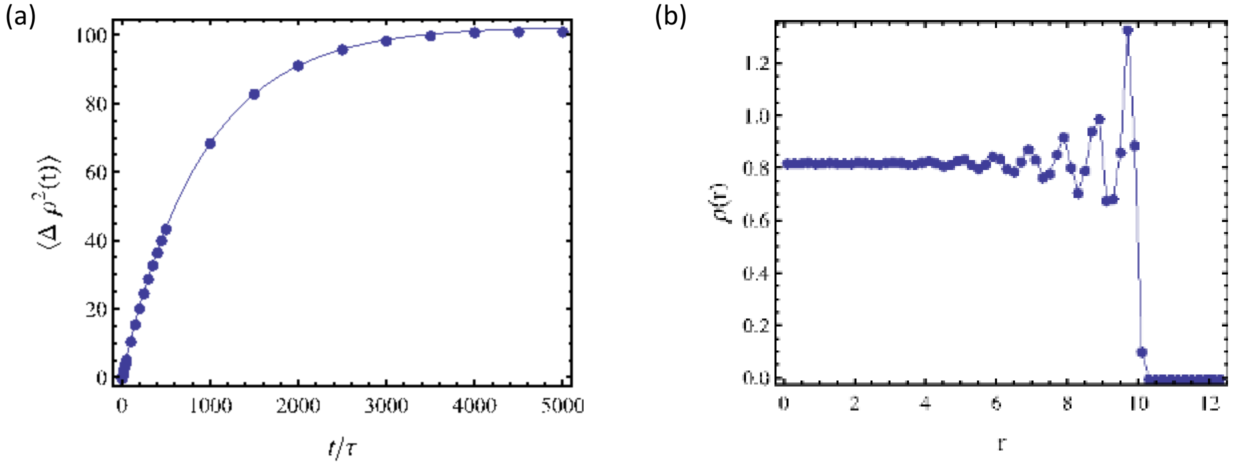

Figure S5. (a) Mean-square displacement along the radial direction of the channel. The solid line is a fit to the data with the functional form of Eq.(S1). (b) Radial density within the nano channel as function of radial distance. The density profile shows some layering structure near the wall. The radial density profile was obtained by averaging the fluid density in cylidrical shells of thickness $0.3\ \sigma$ and length $44.8\ \sigma$. We observed that the fluid density vanishes for a value of $R = 10.1\ \sigma$, which agrees with the value of R obtained from fitting Eq. 1 the radial MSD data.

$$\langle \Delta \boldsymbol{r}_{\perp}^{2}(t)\rangle = R^{2}\left[1-\sum_{n=1}^{\infty}\frac{8}{\lambda_{1n}^{2}(\lambda_{1n}^{2}-1)}e^{-(\lambda_{1n}^{2}/R^{2})D_{\perp}t}\right]. \quad \text{(S1)}$$

Dynamic Monte Carlo Simulations.

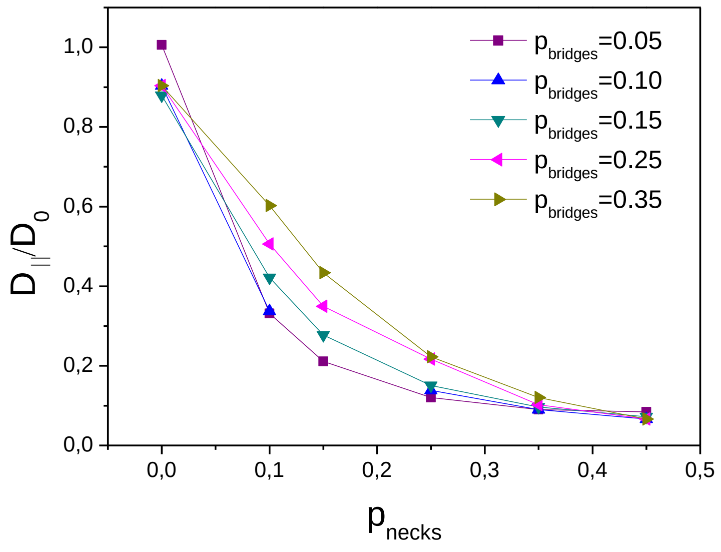

Figure S6. Normalized diffusivity along the channels as a function of the constrictions density p_{necks} for different densities p_{bridges} of the interconnections between adjacent channels indicated in the inset.

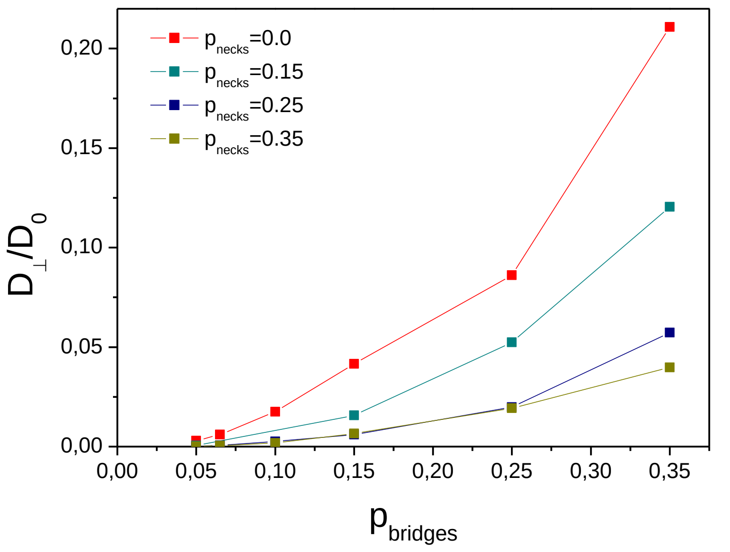

Figure S7. Normalized diffusivity perpendicular to the channel direction as a function of the interconnections density p_{bridges} for different densities of the constrictions p_{necks} as indicated in the inset.
